# A large-scale surveillance revealed that KPC variants mediated ceftazidime-avibactam resistance in clinically isolated *Klebsiella pneumoniae*

**DOI:** 10.1128/spectrum.00258-24

**Published:** 2024-07-03

**Authors:** Sai Qiao, Shaojun Xin, Yufeng Zhu, Feng Zhao, Heng Wu, Jingjing Zhang, Bingyan Yao, Yunsong Yu, Ying Fu, Yan Jiang, Xinyou Xie, Jun Zhang

**Affiliations:** 1 Department of Clinical Laboratory, Sir Run Run Shaw Hospital, School of Medicine, Zhejiang University, Hangzhou, Zhejiang, China; 2 Key Laboratory of Precision Medicine in Diagnosis and Monitoring Research of Zhejiang Province, Sir Run Run Shaw Hospital, Hangzhou, Zhejiang, China; 3 Department of Clinical Laboratory, Huzhou Central Hospital, Affiliated Huzhou Hospital, School of Medicine, Zhejiang University, Huzhou, Zhejiang, China; 4 Department of Clinical Laboratory, Hangzhou Xixi Hospital, School of Medicine, Zhejiang University, Hangzhou, Zhejiang, China; 5 Department of Clinical Laboratory, Zhejiang University Sir Run Run Shaw Alar Hospital, Alar, Xinjiang Uygur Autonomous Region, Xinjiang, Zhejiang, China; 6 Department of Infectious Diseases, Sir Run Run Shaw Hospital, School of Medicine, Zhejiang University, Hangzhou, Zhejiang, China; 7 Key Laboratory of Microbial Technology and Bioinformatics of Zhejiang Province, Hangzhou, Zhejiang, China; 8 Regional Medical Center for National Institute of Respiratory Diseases, Sir Run Run Shaw Hospital, School of Medicine, Zhejiang University, Hangzhou, Zhejiang, China; London Health Sciences Centre, London, Canada

**Keywords:** *Klebsiella pneumoniae*, ceftazidime-avibactam, clinically induced resistance, gene mutation, omega loop

## Abstract

To monitor the resistance rate and gain a deeper understanding of the resistance mechanisms, we conducted over a 2-year surveillance focusing on the *Klebsiella pneumoniae* associated with the clinical usage of ceftazidime-avibactam (CZA) in a teaching hospital. A total of 4,641 *K*. *pneumoniae* isolates were screened to identify the CZA resistance through antimicrobial susceptibility testing. Comprehensive analyses, including homology analysis, conjugation experiments, clone assays, and whole genome sequencing, were furtherly performed on the CZA-resistant strains. In total, four CZA-resistant *K. pneumoniae* (CZA-R-Kp) strains were separated from four patients, in which three of them received CZA treatment during the hospitalization, accounting for a 4% (3/75) resistance development rate of *K. pneumoniae* under CZA stress. All CZA-R-Kp isolates were found to possess variants of *bla*_KPC-2_. The identified mutations included *bla*_KPC-33_, *bla*_KPC-86_, and a novel variant designated as *bla*_KPC-129_, all of which were located in the Ω loop of the KPC enzyme. These mutations were found to impact the amino acid sequence and spatial structure of the enzyme’s active center, consequently affecting KPC carbapenemase activity. This study underscores the importance of active surveillance to monitor the emergence of resistance to CZA, highlighting the need for ongoing research to develop effective strategies for combating antimicrobial resistance. Understanding the mechanisms behind resistance is crucial in maintaining the efficacy of CZA, a vital tool in the battle against multidrug-resistant infections.

**IMPORTANCE:**

As an effective drug for the treatment of carbapenem-resistant *Klebsiella pneumoniae*, ceftazidime-avibactam (CZA) began to develop resistance in recent years and showed an increasing trend. In order to effectively monitor the resistance rate of CZA and understand its resistance mechanism, we monitored *K. pneumoniae* for more than 2 years to find CZA-resistant strains. Through comprehensive analysis of the selected CZA-resistant strains, it was found that all the CZA-resistant strains had mutation, which could affect the activity of KPC carbapenemase. This study highlights the importance of proactive surveillance to monitor the emergence of CZA resistance, which highlights the need for ongoing research to develop effective strategies to combat antimicrobial resistance. Understanding the mechanisms behind resistance is critical to maintaining the effectiveness of CZA, an important tool in the fight against multidrug-resistant infections.

## INTRODUCTION

*Klebsiella pneumoniae* stands as one of the most threatening pathogens in nosocomial infections. The clinical utilization of ceftazidime/avibactam (CZA) for treating carbapenem-resistant *K. pneumoniae* (CRKP) has become widespread (1), yet the increasing drug resistance is a concern for clinicians. Previously, the epidemics of CRKP in China and the USA during 2015 to 2019 were analyzed, revealing an average prevalence of CRKP at 10.9% (ranging from 0.6% to 32.8%) and 4.7% (ranging from 0% to 30%), respectively (2). Currently, there are few drug options for the treatment of CRKP in clinical practice, with CZA emerging as one of the few effective choices (3). Notably, a large proportion of CRKP prevalent in China is KPC producing, making CZA the most suitable treatment for infection of KPC resistance. However, reports of CZA resistance have emerged. Multiple research studies have identified KPC variants, such as KPC-33, KPC-86, KPC-87, and KPC-88 (4), with new mutation variants intermittently reported. The focus of these studies on the mechanism of CZA resistance revolves around KPC variants, which alter the Ω loop structure of KPC carbapenemase, enhancing affinity to ceftazidime while weakening affinity to avibactam, thereby mediating bacterial resistance to CZA (5).

Several previous studies have reported the development of CZA resistance during clinical practice, but the frequency of resistance emergence after CZA treatment remains unclear. Hence, we conducted a long-term study involving prospective clinical monitoring of CZA-resistant *K. pneumoniae*, aiming to explore the mechanism of drug resistance and the rate of resistance development during clinical practice. The results indicate a low incidence of CZA-resistant *K. pneumoniae*, with only four positives for CZA resistance. All four resistant strains exhibited Ω loop point mutations on *bla*_KPC_. Furthermore, a novel variant, designated as *bla*_KPC-129_, was identified in one of our isolates.

## MATERIALS AND METHODS

### Surveillance

Since the approval of CZA in China in 2019, we conducted surveillance for CZA resistance among clinically isolated *Klebsiella pneumoniae* in a 2,400-bed teaching hospital from 1 January 2020 to 31 May 2022, aiming to explore potential resistance with or without CZA treatment. After excluding patient duplication, a total of 4,641 *K*. *pneumoniae* isolates were collected from 1,845 patients, including 75 patients who received CZA treatment during their hospitalization. Matrix-assisted laser desorption ionization time-of-flight mass spectrometry (MALDI-TOF MS, Mérieux, France) was utilized for species identification. Clinical information from all enrolled patients was obtained through electronic medical records.

### Antimicrobial susceptibility testing

Antimicrobial susceptibility testing (AST) was conducted to determine the MICs of 19 antimicrobial agents by using the VITEK Compact 2 system (Mérieux, France), including ampicillin, ampicillin-sulbactam, piperacillin-tazobactam, ceftazidime, cefotetan, ceftriaxone, cefepime, imipenem, ertapenem, aztreonam, ciprofloxacin, gentamicin, amikacin, levofloxacin, trimethoprim/sulfamethoxazole, and tobramycin. The broth microdilution method was employed to determine the MICs of CZA, meropenem, tigecycline, colistin, meropenem-vaborbactam, and imipenem-relebactam. The 2022 EUCAST guideline was used for tigecycline interpretation (http://www.eucast.org) (6). *Escherichia coli* ATCC 25922 served as the quality control. The carbapenemase phenotype was detected using NG-Test CARBA 5 (FOSUN Pharma), followed by PCR and sequencing using universal primers for the *bla*_KPC_ gene (7).

### Pulsed-field gel electrophoresis

Homology analysis of paired isolates, comprising one CZA-resistant isolate matched with one CZA-susceptible isolate selected from the same patient, was performed using pulsed-field gel electrophoresis (PFGE) after digestion with the XbaI restriction enzyme, as previously described (8).

### Clone of the novel *bla*_KPC_ variant

The novel *bla*_KPC-2_ variant was amplified and cloned to verify the CZA-resistant phenotype. Genomic DNA extraction was carried out using the QIAamp DNA Mini Kit (Qiagen, New York, USA) following the manufacturer’s instructions. First, PCR amplification was performed using specific clonal primers (Table S1). Then, we used the TA clone method to insert PCR-amplified gene fragments with single 3′-A overhangs on each end into a plasmid vector containing a complementary 5′-T overhang. After confirmation by sequencing, the fragment of the *bla*_KPC_ variant was then cloned into the pCR2.1 vector using ClonExpress technology (Vazyme, Nanjing, China) following digestion by HindIII and BamHI restriction enzymes. The resulting ligation product was transformed into *E. coli* DH5α competent cells using the heat shock method. Mueller Hinton (MH) plates containing 50 mg/L kanamycin were used for the selection of *E. coli* DH5α positive for pCR2.1-*bla*_KPC_. Colonies growing on the selection plate were confirmed by PCR. The pCR2.1 vector was introduced into *E. coli* DH5α as a control. AST was then conducted to determine the MICs of both *E. coli* DH5α (pCR2.1- *bla*_KPC_) and *E. coli* DH5α (pCR2.1).

### Horizontal transferability of *bla*_KPC_ plasmid

S1-PFGE and southern blotting were used to confirm the location of the *bla*_KPC_ gene, as previously reported (9, 10). Conjugation assays were performed using filter mating, with *bla*_KPC_-positive isolates serving as donors and *E. coli* J53 (azide resistant) as recipients on a filter membrane resting on MH agar without antibiotics. After 18 hours of incubation, the mixed cultures were plated onto MH agar containing ampicillin (100 mg/L) and azide (200 mg/L). Transconjugants were selected after 48 hours of incubation. Plasmids were also extracted from the CZA-resistant *K. pneumoniae* isolates and electro-transformed into *E. coli* DH5α competent cells, followed by selection on plates containing 100 µg/mL ampicillin. Species identification, AST, and PCR confirmation were performed on both conjugants and electro-transformants upon acquisition (11).

### Whole genome sequencing and data analyses

*Klebsiella pneumoniae* isolates demonstrating CZA resistance, along with the corresponding CZA-susceptible isolates collected from the same patient, were chosen for whole genome sequencing (WGS) and subsequent data analyses. Genomic DNA extraction was conducted using the QIAamp DNA Mini Kit (Qiagen, Valencia, CA). WGS was carried out using the Illumina HiSeq X Ten platform (Illumina, San Diego, CA) followed by the MinION device (Oxford Nanopore Technologies Inc., UK). Unicycler v0.4.8 and Prokka 1.11 were employed for generating the complete genome sequence and gene annotation, respectively. Mapping and genome comparison between CZA-resistant and CZA-susceptible isolates were performed using breseq to explore potential mutations (12). The online PubMLST tool was utilized for the detection of multilocus sequence typing (https://pubmlst.org/bigsdb?db=pubmlst_koxytoca_seqdef). Plasmid replicon types were determined using PlasmidFinder and KpVR tools (https://cge.cbs.dtu.dk/services/PlasmidFinder/ and https://db-mml.sjtu.edu.cn/KpVR/). Antimicrobial resistance genes were identified using the ResFinder database with Abricate 0.8 (https://github.com/tseemann/abricate) and BacAnt. The IS Finder (www-is.biotoul.fr/) and oriTfinder (https://tool-mml.sjtu.edu.cn/oriTfinder/oriTfinder.html) were used for plasmid sequence analysis. Graphic maps were generated using the CGView server (http://stothard.afns.ualberta.ca/cgview_server). Sequence comparisons were performed using BLASTn v2.4.0.

## RESULTS

### Surveillance of CZA resistance

During over 2 years of CZA resistance surveillance, a total of 4,641 *K*. *pneumoniae* isolates were collected from 1,845 non-duplicate patients, among which 1,667 isolates from 491 patients were CRKP. Consequently, four CZA-resistant *K. pneumoniae* isolates from distinct patients were obtained, with three of them collected from patients who received CZA treatment during their hospitalization, accounting for a resistance development rate of 4.0% (3/75) of *K. pneumoniae* under CZA stress. Notably, the remaining isolate was obtained from a patient who had not undergone CZA therapy, although other β-lactam antibiotics were administered during the patient’s hospitalization.

### Patients

The four patients from whom CZA-resistant *K. pneumoniae* isolates were collected suffered from severe infectious diseases (Table 1). Among them, three patients are male and one is female, aged between 28 and 69 years, all of whom had a medical history of intensive care unit (ICU) admission. For the three patients who received CZA treatment, multiple antimicrobial agents were administered prior to the isolation of CZA-resistant strains. The duration of CZA treatment ranged from 8 to 33 days. A series of *K. pneumoniae* strains were isolated from each of these four patients, and we selected one of the CZA-susceptible strains identified in each patient before the isolation of the CZA-resistant *K. pneumoniae* for subsequent homolog comparison. The paired strains, which were susceptible to CZA before the CZA-R-KP isolated, were selected in our study and designated as XYJ-CZA-S/-R, GYH-CZA-S/-R, ZZX-CZA-S/-R, and WHY-CZA-S/-R, respectively (Table 2).

**TABLE 1:** Clinical information of patients who had suffered from infection of CZA-resistant CRKP^*a*^

No.	Strain code	Infectious disease	Age	Gender	Ward	Days of CZA usage	Other antimicrobial agents used in treatment
1	XYJ	Pneumonia	28	M	ICU	8	PZT, MEM, PB, TGC
2	GYH	Peritoneal infection	65	M	ICU	10	CAZ, SCF, IPM, VA, LEV, SXT
3	ZZX	Pneumonia	70	M	ICU	33	CRO, SCF, MEM, MXF, LZD, CLA
4	WHY	Urinary tract infection	69	F	ICU	NA	CRO, SCF, IPM, MEM, PZT, LEV, LZD

^*a*^ PZT, piperacillin-tazobactam; MEM, meropenem; PB, polymycin B; TGC, tigencycline; CAZ, ceftazidime; SCF, cefoperazone-sulbactam; IPM, imipenem; VA, vancomycin; LEV, levofloxacin; SXT, sulfamethoxazole-trimethoprim; CRO, ceftriaxone; MXF, moxifloxacin; LZD, linezolid; CLA, clindamycin; F, female; M, male; NA, not applied.

**TABLE 2:** The genotypic and phenotypic characteristics of the four paired isolates and hydrolytic activity of *bla*_KPC_ gene of CZA-R *K. pneumoniae* isolate detected by electro-transformation and TA clone numbers in bold mean resistance^*a*^^,^^*b*^

Strain name	Information of paired isolate			VITEK result (μg/mL)																Broth dilution method (μg/mL)					
	Sample source	Isolation date	*bla*_KPC_ type	AMP	SAM	TZP	CAZ	CTT	CRO	FEP	IMP	ETP	ATM	CIP	GEN	AMK	LEV	SXT	TOB	MEM	TGC	CO	CZA	MEV	IMR
XYJ-CZA-S	Sputum	31 October 2021	*bla*_KPC-2_	**≥32**	**≥32**	**≥128**	**≥64**	**≥64**	**≥64**	**≥64**	**≥16**	**≥8**	**≥64**	**≥4**	**≥16**	1	**≥8**	**≥320**	**8**	**>16**	1	≤0.25	1	2/8	1/4
XYJ-CZA-R	Sputum	8 November 2021	*bla*_KPC-33_	**≥32**	**≥32**	16	**≥64**	**≥64**	**≥64**	**16**	≤1	**≥8**	4	**≥4**	**≥16**	8	**≥8**	80	**8**	2	0.5	≤0.25	**>16**	0.5/8	0.5/4
GYH-CZA-S	Ascitic fluid	18 March 2022	*bla*_KPC-2_	**≥32**	**≥32**	**≥128**	**≥64**	**≥64**	**≥64**	**≥64**	**≥16**	**≥8**	**≥64**	**≥4**	**≥16**	**≥64**	**≥8**	**≥320**	**≥16**	**>16**	0.5	≤0.25	2	1/8	2/4
GYH-CZA-R	Sputum	9 April 2022	*bla*_KPC-129_	**≥32**	**≥32**	**≥128**	**≥64**	**≥64**	**≥64**	**≥64**	2	**≥8**	**≥64**	**≥4**	**≥16**	**≥64**	**≥8**	**≥320**	**≥16**	2	1	≤0.25	**>16**	1/8	0.5/4
ZZX-CZA-S	Sputum	28 March 2022	*bla*_KPC-2_	**≥32**	**≥32**	**≥128**	**≥64**	**≥64**	**≥64**	**≥64**	**≥16**	**≥8**	**≥64**	**≥4**	**≥16**	**≥64**	**≥8**	**≥320**	**≥16**	**>16**	1	≤0.25	2	1/8	1/4
ZZX-CZA-R	Sputum	25 April 2022	*bla*_KPC-86_	**≥32**	**≥32**	**≥128**	**≥64**	**≥64**	**≥64**	**≥64**	≤1	**≥8**	**≥64**	**≥4**	**≥16**	**≥64**	**≥8**	**≥320**	**≥16**	2	0.5	≤0.25	**>16**	1/8	1/4
WHY-CZA-S	Urine	5 March 2022	*bla*_KPC-2_	**≥32**	**≥32**	**≥128**	**16**	**8**	**16**	4	**≥16**	**≥8**	**≥64**	**≥4**	≤1	≤2	**≥8**	40	≤1	**16**	0.5	≤0.25	1	<0.06/8	0.5/4
WHY-CZA-R	Urine	5 May 2022	*bla*_KPC-33_	**≥32**	**16**	8	**≥64**	≤4	**8**	2	≤1	≤0.5	≤1	**≥4**	≤1	≤2	**≥8**	80	≤1	<0.125	0.25	≤0.25	**>16**	<0.06/8	0.25/4
*E. coli* DH5α (pKPC-GYH-CZA-R)				**≥32**	**≥32**	≤4	**≥64**	≤4	8	≤1	≤1	≤0.5	**≥64**	≤0.25	**≥16**	**≥64**	≤0.25	≤20	**≥16**		0.125	≤0.25	8		
*E. coli* DH5α (pKPC-ZZX-CZA-R)				**≥32**	**≥32**	≤4	**≥64**	≤4	8	≤1	≤1	≤0.5	**≥64**	≤0.25	**≥16**	**≥64**	≤0.25	≤20	**≥16**		0.125	≤0.25	2		
*E. coli* DH5α (pCR2.1-blaKPC129)				8	4	≤4	**≥64**	≤4	≤1	≤1	≤1	≤0.5	≤1	≤0.25	≤1	4	≤0.25	≤20	≤1		0.125	0.5	**32**		
*E. coli* DH5α (pCR2.1)				8	4	≤4	**16**	≤4	≤1	≤1	≤1	≤0.5	≤1	≤0.25	≤1	4	≤0.25	≤20	≤1		0.25	≤0.25	4		
*E. coli* DH5a				≤2	≤2	≤4	≤1	≤4	≤1	≤1	≤1	≤0.5	≤1	≤0.25	≤1	≤2	≤0.25	≤20	≤1		0.25	0.25	<0.125		

^*a*^ AMP, ampicillin; SAM, ampicillin-sulbactam; TZP, piperacillin-tazobactam; CAZ, ceftazidime; CTT, cefotetan; CRO, ceftriaxone; FEP, cefepime; IMP, imipenem; ETP, ertapenem; ATM, aztreonam; CIP, ciprofloxacin; GEN, gentamicin; AMK, amikacin; LEV, levofloxacin; SXT, trimethoprim-sulfamethoxazole; TOB, tobramycin; MEM, meropenem; TGC, tigecycline; CO, colistin; CZA, ceftazidime-avibactam; MEV, meropenem-vaborbactam; IMR, imipenem-relebactam.

^*b*^ Numbers in bold mean resistance.

### AST results

The AST results confirmed that all paired isolates were pan-drug resistant, displaying resistance to β-lactams, carbapenems, fluoroquinolones, aminoglycosides, and tetracyclines. Additionally, the CZA-resistant isolates exhibited varying degrees of resistance to cefepime, amikacin, trimethoprim/sulfamethoxazole, and tobramycin. Notably, apart from CZA, the resistance pattern was generally consistent for each paired strain, although some differences were observed, particularly in susceptibility to carbapenems. All four CZA-resistant strains showed reversed susceptibility to carbapenems, such as meropenem with MIC 2 or <0.125 µg/mL compared with CZA-susceptible strains. The phenomenon of regaining carbapenem susceptibility due to the acquisition of CZA resistance was consistent with previous reports (13). Furthermore, all paired strains demonstrated sensitivity to tigecycline, colistin, meropenem-vaborbactam, and imipenem-relebactam (Table 2). The MICs of CZA for the four CZA-resistant strains were greater than 16 µg/mL, indicating a high level of resistance to this antibiotic combination (Table 2).

### Homology analysis

Each paired strains exhibited the same PFGE pattern, with fewer than three different bands observed, indicating that the two isolates in each pair originated from the same clone (Fig. S1A). Genome analysis of each paired strains also revealed the same ST type, with three pairs identified as ST11 and the remaining pairs identified as ST716, and the latter one is rarely reported for CZA resistance dissemination. The SNP analysis of each paired strains demonstrated the most detailed genome variants. Three out of four pairs of strains exhibited extremely few SNP differences, all fewer than 10 SNP differences, except for the pair ZZX-CZA-S/-R. However, the differences between each pair of strains did not occur in the known antibiotic resistance genes, except for the *bla*_KPC-2_ gene.

### *bla*_KPC_ mutations contribute to CZA resistance

We conducted a comparison of changes in *bla*_KPC_ genes between CZA-susceptible and CZA-resistant isolates from the same patient, revealing the contribution of KPC mutations to CZA resistance. Using NG-Test CARBA 5, all CZA-susceptible isolates tested positive for the carbapenem phenotype, while the corresponding CZA-resistant isolates tested negative (Fig. S2). The PCR and sequencing results showed that all four CZA-susceptible isolates harbored the *bla*_KPC-2_ gene, whereas the paired CZA-resistant isolates were positive for *bla*_KPC-33_ (two isolates), *bla*_KPC-86_, and a novel *bla*_KPC_ allele designated as *bla*_KPC-129_ in this study, respectively (Table 2). According to the alignment results of the amino acid sequences of KPC carbapenemase, XYJ-CZA-R and WHY-CZA-R exhibited a point mutation compared with the KPC-2 protein, resulting in a change from D to Y at site 179 of the Ω loop. ZZX-CZA-R exhibited a D to G point mutation at site 179 and expressed a KPC-86 phenotype. Furthermore, GYH-CZA-R exhibited a novel N to H mutation at site 170 of the Ω loop, named KPC-129. KPC-129 represents the first report of this mutation phenotype in clinically CZA-resistant KPC strains (Fig. S3).

### *bla*_KPC_ plasmids and their locations

We employed S1-PFGE to investigate the presence of plasmids in each of the isolated strains carrying *bla*_KPC_ resistance determinants. Southern blotting experiments confirmed that *bla*_KPC_ genes among the four paired strains were indeed located on plasmids, with sizes ranging from 54.7 kb to 216.9 kb (Fig. S2B). In the strains XYJ-R and WHY-R, the *bla*_KPC-33_ gene was located on a plasmid of approximately 78.2 kb. The *bla*_KPC-129_ gene was detected in the GYH-R strain, associated with a plasmid of approximately 104.5 kb, while the *bla*_KPC-86_ gene was identified in the ZZX-R strain, linked to a plasmid of approximately 138.9 kb (Fig. S2B).

### Hydrolytic activity of the *bla*_KPC_ gene of the CZA-R *K. pneumoniae* isolate

Several attempts of filter mating experiments to transfer CZA-resistant plasmids to *E. coli* J53 recipients failed, indicating that the plasmids in these strains were incapable of being transferred by conjugation. Since the CZA resistances mediated by KPC-86 and KPC-129 carbapenemases were not reported previously, *E. coli* DH5α transformants containing pKPC-GYH-CZA-R and pKPC-ZZX-CZA-R were furtherly constructed. Both plasmids were found to mediate increases in the MICs of β-lactams, including ampicillin, ceftazidime, ampicillin-sulbactam, piperacillin-tazobactam, ceftazidime-avibactam, aztreonam, gentamicin, and amikacin. As a novel carbapenemase, KPC-129 was also shown to confer resistance to both ceftazidime and CZA, as revealed by TA clone experiments (Table 2).

### Genomic analysis of *bla*_KPC_ plasmids

**Fig 1:**
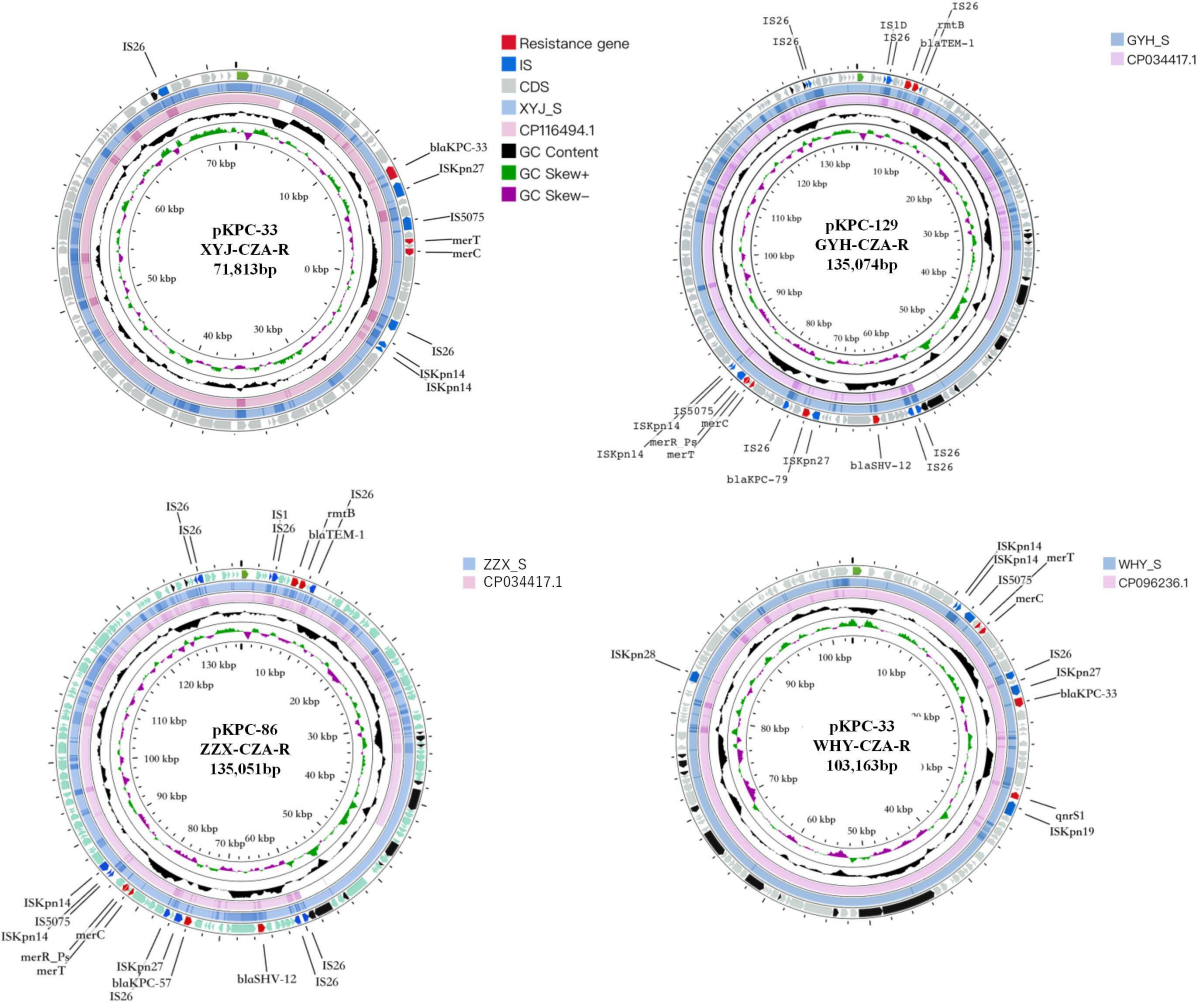
Characteristics and alignment of plasmids containing CZA-resistant genes. Circular map of the KPC-R and comparative genomics analysis with its similar plasmids. Starting from the center: (i) GC skew (G−C/G+C), with a positive GC skew toward the inside and a negative GC skew toward the outside. (ii) GC content of pNDM4-191773 with an average of 54.03% (used as reference). (iii) pNDM4-191773 plasmid sequence (CP116494.1). (iv) Gene annotation. Red, antibiotic resistance; gray, transfer conjugation; sea-blue, insertion sequence; gray-blue, the relative KPC-S strain plasmid gene sequence. Arrowhead indicates the direction of transcription. A scale of 1,000  bp is attached to the corner.

WGS was performed to analyze the genomes of the plasmids isolated from the four paired *K. pneumoniae* isolates. As depicted in Fig. 1, the presence of identical genetic organization regions suggested a conservative structure among the four CZA-resistant isolates compared with their paired CZA-sensitive isolates. In the XYJ-CZA-R and WHY- CZA-R strains, the resistance gene *bla*_TEM-1B_ was located downstream of IS*Kpn27*. Additionally, the WHY-R strain exhibited the presence of a *qnrS1* resistance gene downstream of IS*Kpn19*. The ZZX-CZA-R and GYH-CZA-R isolates showed resistance genes, including *bla*_TEM-1_, *rmtB*, and *bla*_SHV-12_, flanked by IS*26*, which differed from the presence of *bla*_KPC-57_ and *bla*_KPC-79_ in relative proportions. Furthermore, all four CZA-resistant strains were found to exhibit mercury resistance genes of *merT* and *merC*. These results collectively suggest that mutations in plasmid resistance genes may induce amino acid substitutions, which could subsequently influence the formation and function of the Ω loop of KPC variants, thereby affecting the hydrolyzation function of KPC enzymes to CZA.

## DISCUSSION

We conducted a surveillance spanning over 2 years, focusing on clinically isolated *K. pneumoniae* strains to monitor the CZA-R-KP strains. All four identified strains were found to possess a *bla*_KPC-2_ variant, including *bla*_KPC-33_, *bla*_KPC-86_, and a novel variant designated as *bla*_KPC-129_. The observed resistance rate of *K. pneumoniae* was lower than the rate reported by CHINET 2023 (China Antimicrobial Surveillance Network), which reported that the CZA resistance rate for CRKP is around 11.0%. These data were based on antimicrobial susceptibility testing *in vitro* of 16,125 CRKP isolates collected from 73 hospitals across China (available from http://www.chinets.com/Data/AntibioticDrugFast). We attribute this lower rate of CZA resistance to differences in drug application choices resulting from single-center studies and variations in antibiotic resistance patterns within large urban populations.

Although CZA has not been extensively used in clinical practice for an extended period, reports have emerged indicating the occurrence of drug resistance, particularly in organisms such as *K. pneumoniae* (14, 15). For instance, there have been reports of a clinical isolate of *K. pneumoniae* demonstrating high-level resistance to CZA, attributed to the production of KPC-53 due to a Leu167Glu168 duplication in the Ω loop and subsequent loss of carbapenemase activity (16). In our study, three patients received CZA treatment, suggesting that the development of drug resistance may not necessarily correlate with the duration of drug use and therefore underscoring the importance of cautious and supervised use throughout the treatment process. We also report a case where CZA resistance occurred in a patient with no history of CZA usage but had been treated with various antibiotic drugs, particularly ceftazidime. The patient received ceftazidime treatment for 28 days before developing CZA resistance, prompting the hypothesis that the introduction of ceftazidime might have induced CZA resistance (17). Previous research has shown that CRKP could acquire resistance to CZA and tigecycline *in vivo* under the exposure to β-lactam antibiotics and TGC (18).

Studies have reported various mechanisms of CZA resistance, including mutations within the coding region of *bla*_KPC-3_(19, 20), deficiency of OmpK35 porin (21), mutations within *bla*_KPC-2_ (22, 23), and porin deficiency associated with a higher *bla*_KPC_ copy number (24).

The active site within the Ω loop plays a crucial role in the catalytic activity of class A β-lactamases, located adjacent to the catalytic residue S70. The Ω loop also contains a position for a catalytic water molecule necessary for the diacylation reaction (25). Within this loop, residues LEU162-ASP179 are present, forming part of the active site of KPC-2 containing ASN170 and ASP179 (Fig. S4). However, the conversion of amino acid types, resulting in HIS170 and GLY179 in KPC-129 and KPC-86, respectively, has been found to cause ceftazidime resistance. Although similar mechanisms of *bla*_KPC_ mutations have been reported, such as *bla*_KPC-2_, *bla*_KPC-33_, and *bla*_KPC-86_, our research introduces a novel mutation named *bla*_KPC-129_. KPC-129 represents a newly discovered mutation phenotype isolated from the GYH-CZA-R strain, featuring a mutation at site 170 of the Ω loop.

In previous studies, CZA-R strains demonstrated collateral sensitivity to β-lactam antibiotics. This suggests that the mutations in the KPC-2 and KPC-3 β-lactamase sequences responsible for CZA resistance can also affect susceptibility to other β-lactam antibiotics (26). In our research, a similar phenomenon was observed, where the development of CZA resistance in *K. pneumoniae* strains resulted in the regaining of sensitivity to several antibiotics like imipenem and meropenem. On the one hand, most clinical laboratories may not test or report ceftazidime-avibactam resistance if carbapenem is tested susceptible. The selection of this compound preparation requires caution. On the other hand, this revival of drug sensitivity may assist in selecting appropriate drugs for treating *K. pneumoniae* infections. From a clinical perspective, the shift in drug resistance phenotype during our treatment with CZA underscores the importance of promptly assessing changes in the *in vitro* susceptibility of the isolates and adjusting antibiotics as needed, particularly for patients receiving CZA. The mechanism underlying this susceptibility change may lie in the protein structural alterations caused by carbapenemase mutations, which alter the hydrolysis profile of the enzyme toward antibiotics. When the variant exhibits enhanced hydrolytic activity toward one antibiotic, it may imply a decreased hydrolytic activity toward another antibiotic. For researchers, exploring the mechanisms could lead to a deeper understanding of the resistance mechanisms of KPC.

In summary, our study presented four clinical cases of *bla*_KPC_ mutants in CZA-resistant isolates, notably including a novel mutation named KPC-129. Given the relatively high incidence of CZA usage in treating *K. pneumoniae* infections, caution should be exercised in the prescription and monitoring of CZA throughout the entire course of treatment. This study underscores the significance of active surveillance to monitor the emergence of resistance to CZA, emphasizing the necessity for further research to combat antimicrobial resistance.

## Data Availability

The nucleotide sequences reported in this study have been submitted to the GenBank database with the BioProject ID numbers PRJNA889558, PRJNA889999, PRJNA890402, PRJNA892659, and PRJNA892718.

## SUPPLEMENTAL MATERIAL

Supplemental material spectrum.00258-24-s0001.docx Fig. S1 to S4; Tables S1 and S2.

ASM does not own the copyrights to Supplemental Material that may be linked to, or accessed through, an article. The authors have granted ASM a non-exclusive, world-wide license to publish the Supplemental Material files. Please contact the corresponding author directly for reuse.
