# Correction for Qiao et al., “A large-scale surveillance revealed that KPC variants mediated ceftazidime-avibactam resistance in clinically isolated *Klebsiella pneumoniae*”

**DOI:** 10.1128/spectrum.02569-24

**Published:** 2024-10-30

**Authors:** Sai Qiao, Shaojun Xin, Yufeng Zhu, Feng Zhao, Heng Wu, Jingjing Zhang, Bingyan Yao, Yunsong Yu, Ying Fu, Yan Jiang, Xinyou Xie, Jun Zhang

## AUTHOR CORRECTION

Volume 12, no. 8, e00258-24, https://doi.org/10.1128/spectrum.00258-24. Page 1, box: "Address correspondence" should read as shown in this author correction.

