# Supplementary material for: A large-scale surveillance revealed that KPC variants mediated ceftazidime-avibactam resistance in clinically isolated Klebsiella pneumoniae
Source: Microbiol Spectr. 2024 Jul 3;12(8):e00258-24. doi: 10.1128/spectrum.00258-24 (PMC11302327; doi:10.1128/spectrum.00258-24)
Supplement: Supplemental material — Fig. S1 to S4; Tables S1 and S2. [file spectrum.00258-24-s0001.docx]

Supplemental Table 1 PCR amplification primer sequences.

| Name | Sequences |
| --- | --- |
| TA-KPC-129-F | 5’- gat tac gcc aag ctt ggt acc TGT TTA TTT TTC TAA ATA CAT TCA AAT ATG TAT C-3’ |
| TA-KPC-129-R | 5’-gcg gcc gtt act agt gga tcc CGC CGC GCG CCG CAA GAT-3’ |

Supplemental Table 2. Genomic information of CZA-R *K. pneumoniae* strains reported in our study.

| Name of isolate | Location | Length (bp) | MLST type | *Inc* type | ARGs | Transposon | *In* |
| --- | --- | --- | --- | --- | --- | --- | --- |
| XYJ-CZA-R | Chromosome | 5375061 | ST 11 | / | blaSHV-182, aadA2, sul1, fosA6 | ND | ND |
|  | Plasmid 1 | 215492 | / | *Inc*FIB(K)(pCAV1099-114), *Inc*HI1B(pNDM-MAR) | ND | ND | ND |
|  | Plasmid 2 | 95599 | / | *Inc*FIB(AP001918), *Inc*FII | blaTEM-1, aac(3)-IId, dfrA17, aadA5, sul1 | ND | ND |
|  | Plasmid 3 | 95564 | / | ND | ND | ND | ND |
|  | Plasmid 4 | 71813 | / | *Inc*FII(pHN7A8), *Inc*R | blaKPC-33 | *Tn*6292(3) | ND |
|  | Plasmid 5 | 11970 | / | ColRNAI | ND | ND | ND |
|  | Plasmid 6 | 5596 | / | Col(pHAD28) | ND | ND | ND |
| GYH-CZA-R | Chromosome | 5517133 | ST 11 | / | blaSHV-182, emrD, fosA6 | *Tn*602 | ND |
|  | Plasmid 1 | 219800 | / | *Inc*FIB(K)(pCAV1099-114)，*Inc*HI1B(pNDM-MAR) | ND | ND | ND |
|  | Plasmid 2 | 135074 | / | *Inc*FII(pHN7A8), *Inc*R | blaTEM-1, blaSHV-12, blaKPC-129, rmtB1 | *Tn*6292, Tn21 | ND |
|  | Plasmid 3 | 87100 | / | ND | blaLAP-2, qnrS1, dfrA14, sul2, catA2, tet(A) | *Tn*6292 | ND |
|  | Plasmid 4 | 41419 | / | ND | ND | ND | ND |
| ZZX-CZA-R | Chromosome | 5512187 | ST 11 | / | blaSHV-182, emrD, fosA6 | *Tn*2012, *Tn*2003, Tn6292 | ND |
|  | Plasmid 1 | 219796 | / | *Inc*FIB(K)(pCAV1099-114)*, Inc*HI1B(pNDM-MAR) | *ter* | *Tn*602 | ND |
|  | Plasmid 2 | 135051 | / | *Inc*FII(pHN7A8); IncR | blaTEM-1, blaSHV-12, blaKPC-86, rmtB1 | *Tn*6292, *Tn*21, *Tn*602 | ND |
|  | Plasmid 3 | 87095 | / | ND | blaLAP-2, qnrS1, dfrA14, sul2, catA2, tet(A) | *Tn*6292 | *In*718 |
| WHY-CZA-R | Chromosome | 5228789 | ST 716 | / | emrD, oqxB26, blaSHV-27, fosA | *Tn*2003 | ND |
|  | Plasmid 1 | 135518 | / | *Inc*FIB(K)(pCAV1099-114), *Inc*HI1B(pNDM-MAR) | dfrA14, qnrB1 | *Tn*602, *Tn*6292 | *In*805 |
|  | Plasmid 2 | 103163 | / | ND | blaKPC-33, qnrS1 | *Tn*602, *Tn*21, *Tn*6292 | ND |
|  | Plasmid 3 | 3631 | / | Col440I | ND | ND | ND |

a, Incompatibility type of plasmid; b, Integron; ND, not detected.


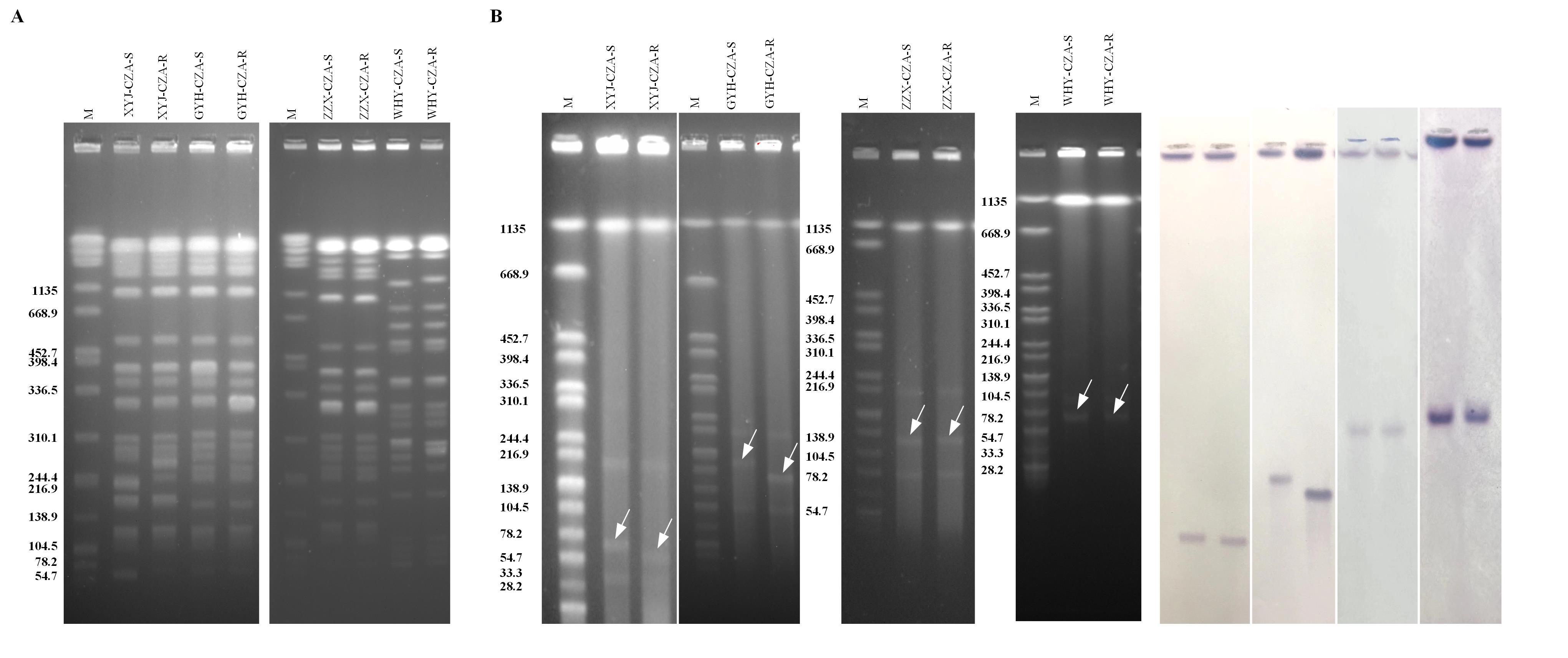


Supplemental Figure 1. Homology analysis based on PFGE and Southern blot analysis on KPC carbapenamase

A. PFGE analysis of four paired isolates of *K. pneumoniae*. In order to generate diagnostic genomic DNA fragmentation fingerprints, genomic DNA from each of the *K. pneumoniae* isolates was digested using XbaI and subjected to pulsed-field gel electrophoresis. DNA fingerprints were revealed by Gel Red staining.

B. DNA fingerprint of S1 enzyme digested plasmid DNA of KPC from clinical isolates stained with ethidium bromide. Lanes 1, 4, 7, 10 were *Salmonella Braenderup* H9812 (molecular weight marker); lane 2-3, 5-6, 8-9, 11-21 were *K. pneumoniae* plasmid isolated from XYJ, GYH, ZZX and WHY relatively; lanes 13-20 were autoradiogram of gel A showing plasmids carrying the *bla*_KPC_ gene correspondent to four paired isolates.


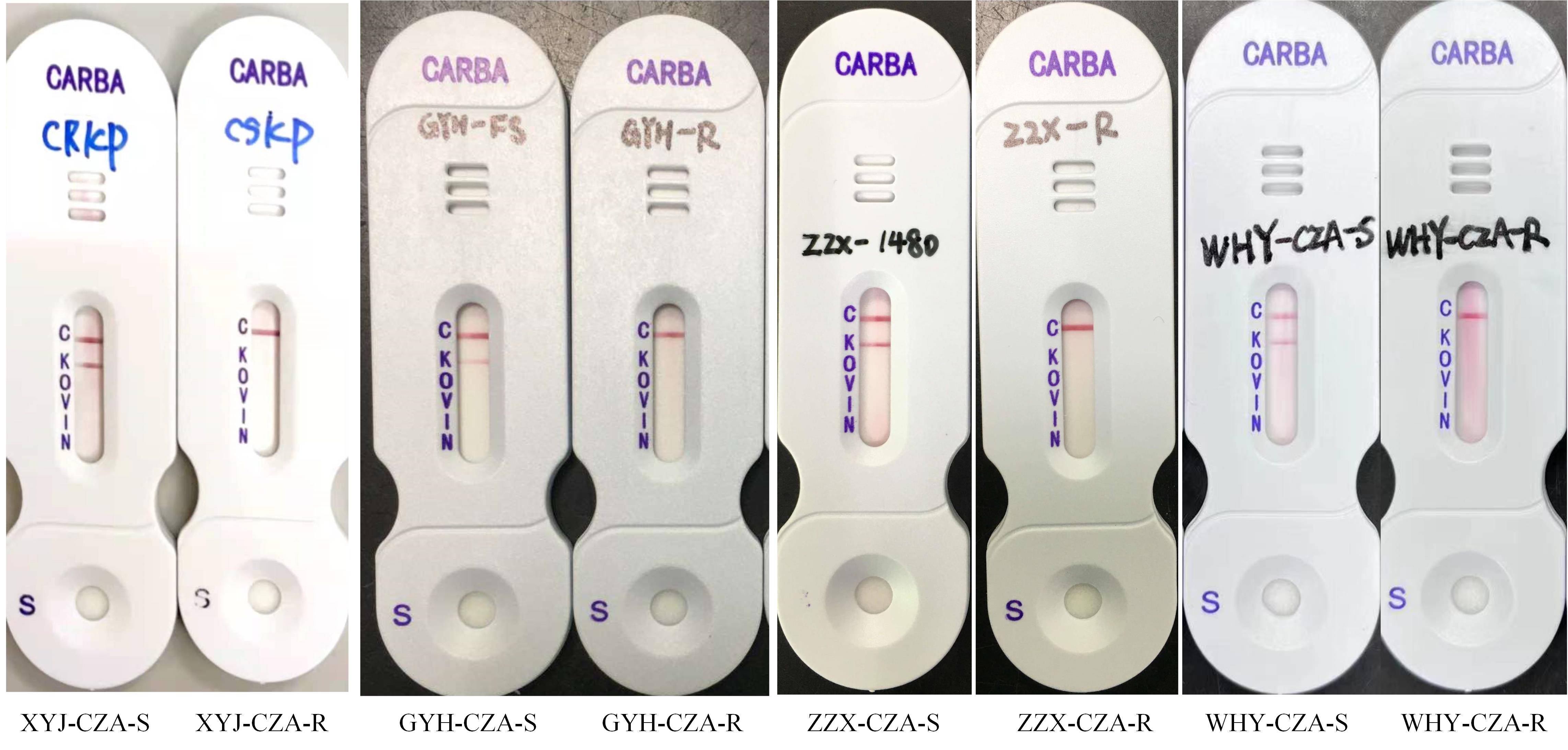


Supplemental Figure 2. Detection results of carbapenemase on paried *K. pneumoniae* isolates

C, control; K, KPC carbapenemase; O, OXA-48-like carbapenemase; V, VIM carbapenemase; I, IMP carbapenemase; N, NDM carbapenemase. The method is based on NG-Test® CARBA 5


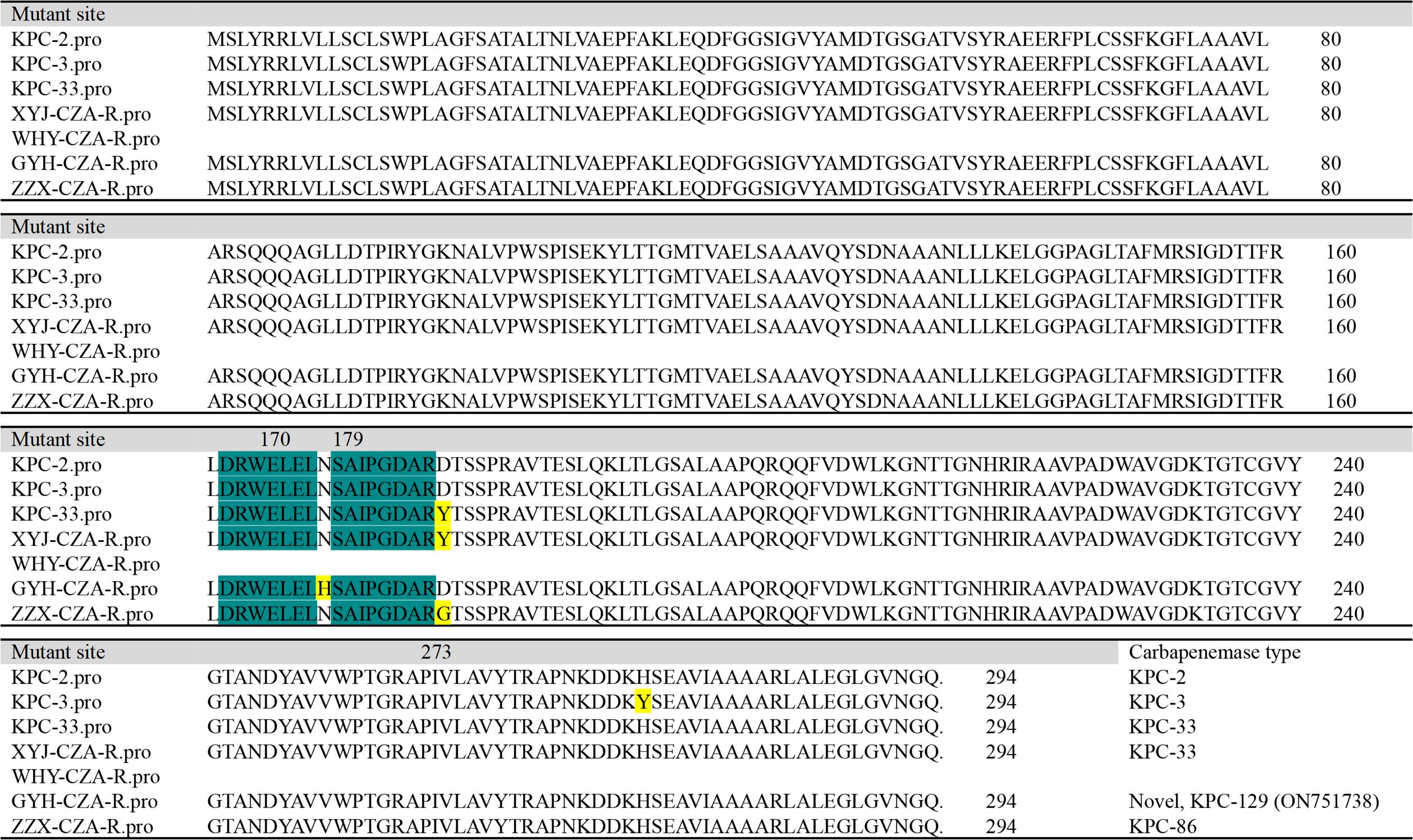


Supplemental Figure 3. Alignment of amino acid sequences of KPC carbapenemase mutants.

The area in color is the place where the differences in sequence among different KPC carbapenemase happened. The sequence in green was the same sequence within the Ω loop, while in yellow was the amino acid with difference.


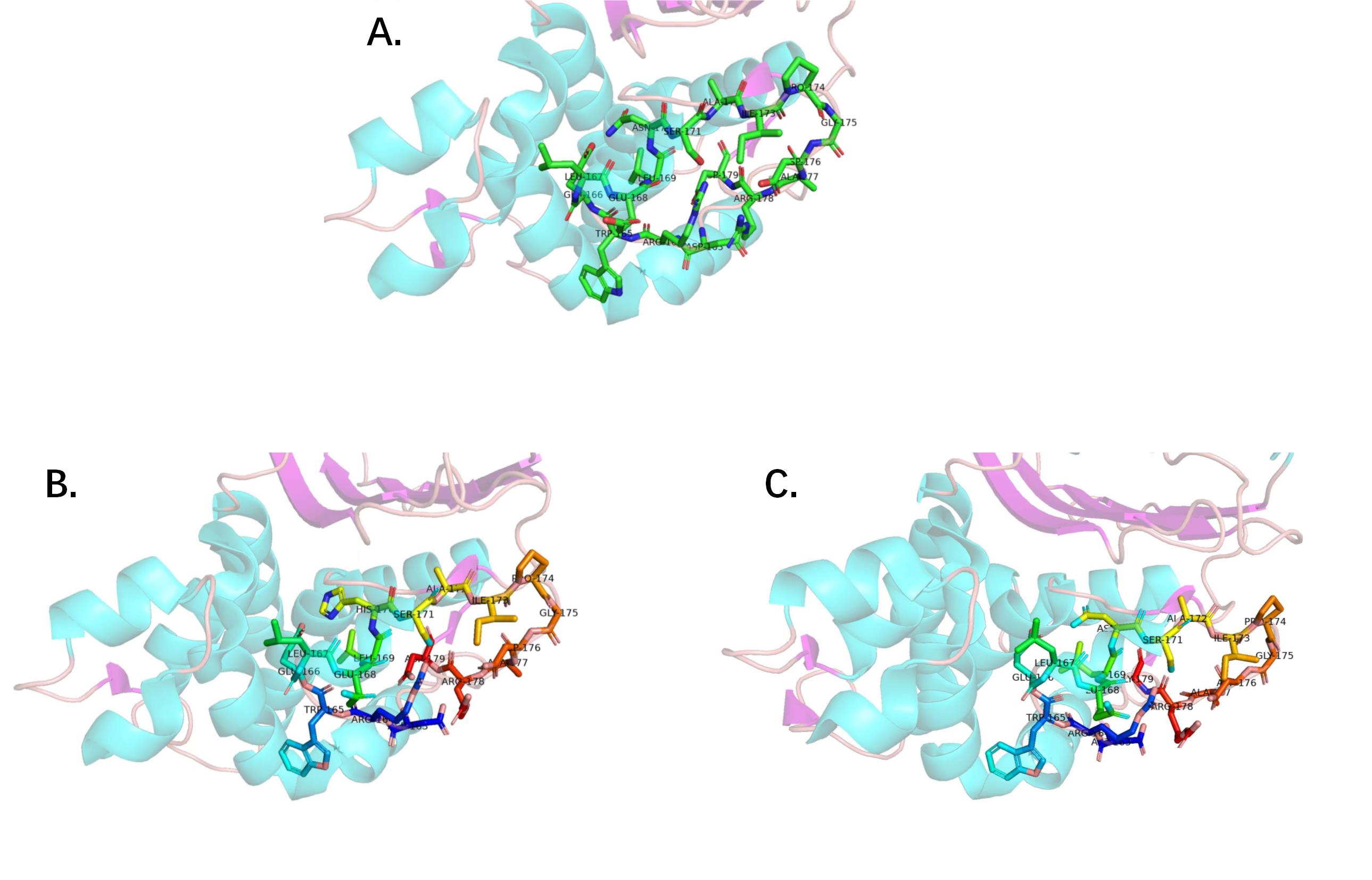


Supplemental Figure 4. Protein crystal structure prediction of CZA-R carbapenemases, KPC-129 and KPC-86

A. The high-resolution CZA-R carbapenemases structure is shown in blurry cyan and violet with the Ω loop depicted in lightful green and blue. The amino acid in the Ω loop is labeled in format as abbreviation-number. B. The predicted protein crystal structure of KPC-129 with a HIS-170 variation comparing to wide-type. C. The predicted protein crystal structure of KPC-86 with a GLY-179 variation different from wide-type.
